# Thrombectomy Versus Anticoagulation: A Case of Pregnancy Complicated by an Extensive Pulmonary Embolism

**DOI:** 10.7759/cureus.107200

**Published:** 2026-04-16

**Authors:** Nicolas Balbi Caruso, Emily Koe, David Majdalany, Linda R Chambliss

**Affiliations:** 1 Department of Obstetrics and Gynecology, Creighton University, Phoenix, USA; 2 Department of Cardiology, Mayo Clinic, Scottsdale, USA

**Keywords:** anticoagulation in pregnancy, high-risk pregnancy, protein s deficiency, pulmonary embolism in pregnancy, severe pre-eclampsia

## Abstract

Pulmonary embolism remains a well-known and life-threatening complication of pregnancy. Due to the variation in presentation and infrequency of this disease, despite serious potential fetal and maternal complications, there is little data available to guide its management in pregnancy. This case highlights the importance of early detection of the condition and the benefit of an interdisciplinary approach to treatment. This case also demonstrates that in the absence of right heart strain, anticoagulation alone may be sufficient management for large pulmonary emboli.

## Introduction

Venous thromboembolism (VTE), which includes both deep vein thrombosis (DVT) and pulmonary embolism (PE), remains a well-known complication of pregnancy, with pregnant women having a 4.0-4.6 times higher risk compared to non-pregnant women [1].

Although the incidence of PE in pregnant patients is quite low at one in 1000 [2] compared to DVT, it accounts for 9-11% of pregnancy-related deaths in the United States [1,3]. The American Heart Association (AHA) and European Society of Cardiology (ESC) categorize PE severity into three main categories, though with different risk-stratification terminology, based on the presence or absence of hemodynamic changes, right ventricular (RV) heart strain, and risk factors for PE [4]. However, standard PE management protocols are challenging to apply in pregnancy due to diagnostic limitations given physiologic changes in pregnancy, restricted anticoagulant options, and the need to balance both maternal and fetal safety [5,6].

The infrequency of the disease, range in presentation and severity, and the lack of specific guidelines make PE in pregnancy difficult to manage. We aim to demonstrate decision-making for extensive PE without RV strain in a high-risk obstetric patient and how multidisciplinary input shapes care.

## Case presentation

A 31-year-old female patient, G2P0101 at 30 weeks and three days, was transported from an outside hospital for chronic hypertension with superimposed severe preeclampsia by blood pressure (BP) criteria. Her pregnancy was complicated by type 2 diabetes mellitus (T2DM) with suboptimal glycemic control, a prior preterm classical cesarean section due to severe pre-eclampsia, a body mass index (BMI) of 42, and a history of recurrent thromboembolisms, including a prior PE and three DVTs. Her prior pregnancy was complicated by a postpartum DVT. She had a thrombectomy and an inferior vena cava (IVC) filter when she had the prior PE. During this pregnancy, she was on enoxaparin 120 mg daily for VTE prophylaxis. 

She had presented to the outside hospital with a complaint of bilateral lower extremity edema and was noted to have sustained severe BP treated with multiple doses of both hydralazine and labetalol and given magnesium for seizure prophylaxis. 

Upon arrival to labor and delivery, her BP was 221/101 mmHg, pulse 93 beats per minute, respiratory rate 28 breaths per minute, and an O2 saturation of 90% on room air. She required 10 L of oxygen via a non-rebreather to maintain a saturation above 94%. On a physical exam, the patient appeared ill with two-plus pitting edema bilaterally in her lower extremities. The fetal heart rate was reactive on the non-stress test. Her hypertension was treated with 20 mg of IV labetalol and 10 mg of immediate-release nifedipine, and the magnesium was continued. Her labs were significant for elevated B-type natriuretic peptide (BNP), urine-protein-creatinine ratio (UPCR), uric acid, and lactate dehydrogenase (LDH), as shown in Table 1.

**Table 1:** Laboratory analyses BNP: B-type natriuretic peptide; UPCR: urine-protein-creatinine ratio; LDH: lactate dehydrogenase; PT: prothrombin; PTT: partial thromboplastin time; INR: international normalised ratio; AST: aspartate aminotransferase; ALT: alanine transaminase

Parameter	Patient Value	Units	Reference values	Hospital Day
BNP	445	pg/mL	0 - 125	1
UPCR	28,737	mg/g	10 - 107	1
Uric acid	7.0	mg/dL	2.5 - 6.2	1
LDH	259	U/L	120 - 246	1
Hemaglobin	13.2	g/dL	11.6 - 14.8	1
Hematocrit	37.8	%	34.4 - 42.8	1
Platelets	324	1000/µL	141 - 464	1
Troponin	0.020	ng/mL	0.000 - 0.034	2
PT	9.9	seconds	9.4 - 12.5	2
PTT	29.9	seconds	25.1 - 36.5	2
INR	0.9	N/A	0.9 - 1.1	2
AST	34	U/L	14 - 36	1
ALT	29	U/L	4 - 35	1
Creatinine	0.45	mg/dL	0.52 - 1.04	1

**Figure 1:**
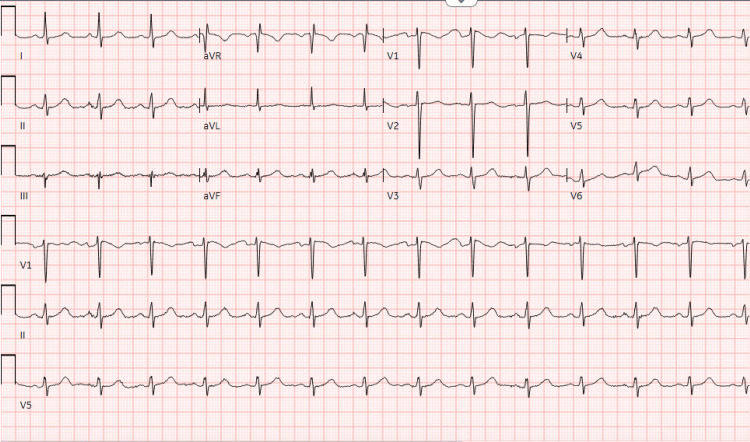
Normal electrocardiogram reading with normal sinus rhythm.

Her records showed a positive lupus anticoagulant, low to normal antithrombin III, and a negative prothrombin gene mutation. An electrocardiogram (EKG) showed a normal sinus rhythm with no evidence of myocardial ischemia (Figure 1).

**Figure 2:**
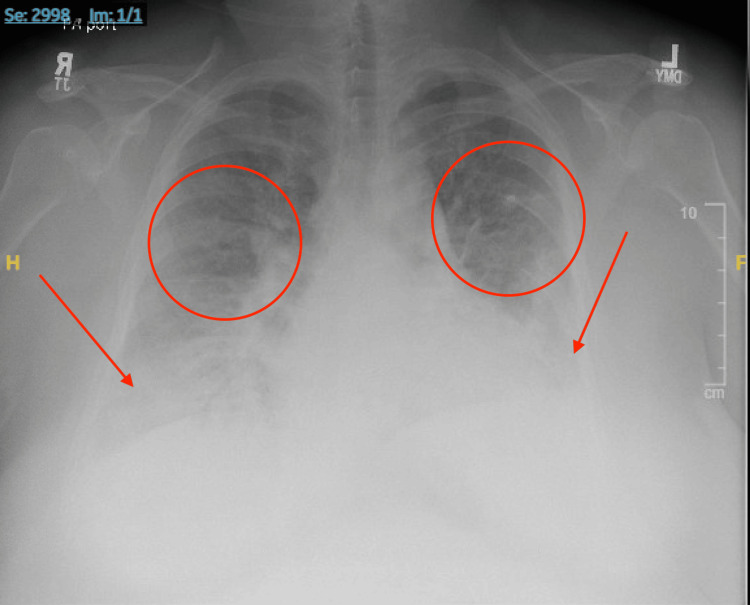
Chest radiograph anterior-posterior view showing perihilar and bibasilar interstitial and airspace opacities (red circles) with small bilateral pleural effusions (red arrows), concerning for pulmonary edema.

Lower extremity dopplers were negative for a DVT. A chest radiograph showed perihilar, bibasilar interstitial and airspace opacities with small bilateral pleural effusions, consistent with pulmonary edema (Figure 2).

**Figure 3:**
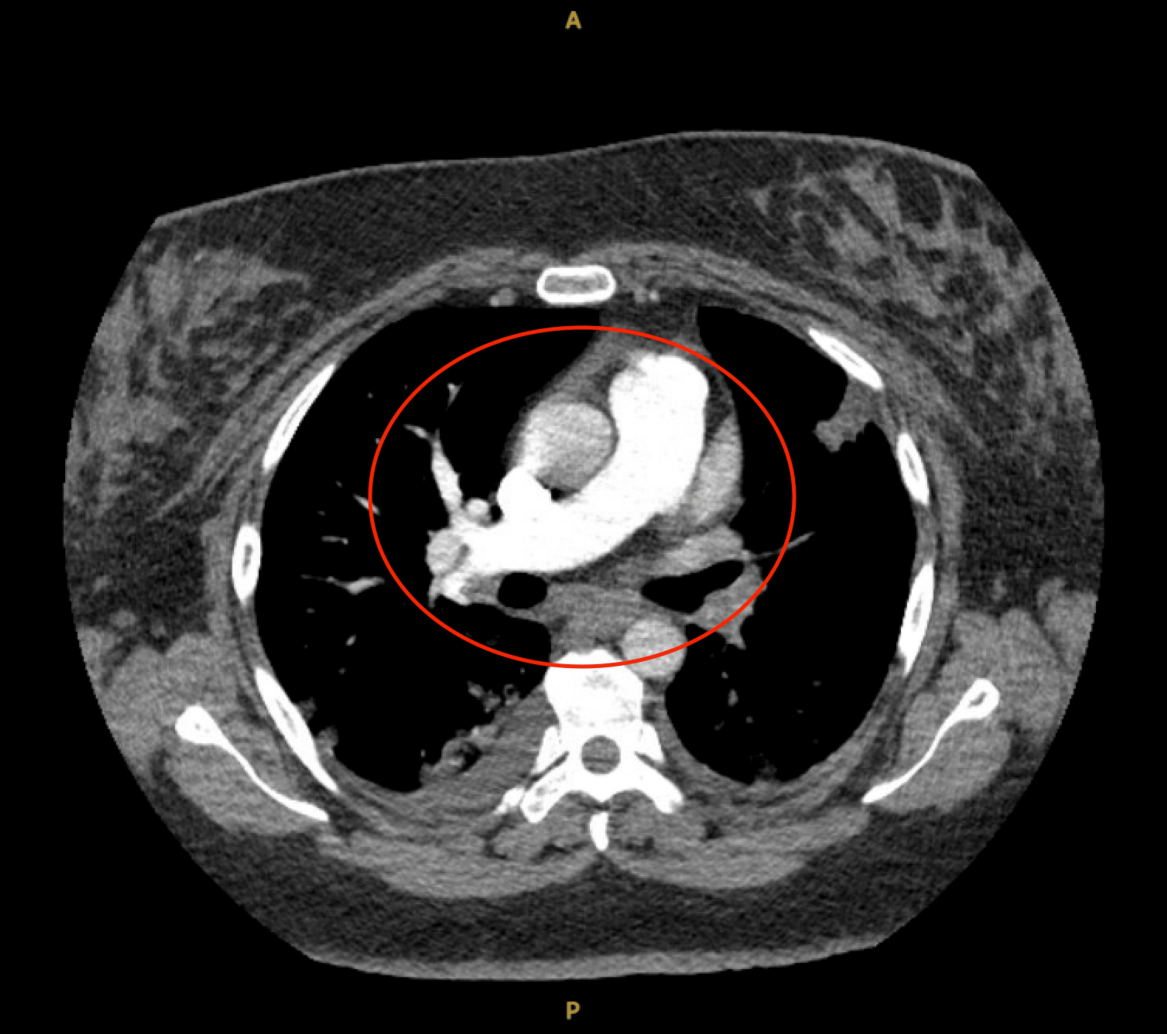
CT angiogram of the chest (axial) showing extensive involvement (noted within the red circle) of the left main pulmonary artery with complete opacification of all distal arterial segments on the left side, including interlobar and subsegmental arteries.

A spiral computed tomography (CT) revealed an extensive PE with total occlusion of the left main pulmonary artery and all distal pulmonary arteries (Figure 3).

The patient's cardiac echocardiogram (ECHO) did not show right heart strain, and right arterial pressure (RAP) was found to be between 0 and 5 mm. The ECHO showing normal right ventricular size and function, without right heart strain, is shown in Video 1.

**Video 1:** Echocardiogram showing normal right ventricular size and function, without right heart strain.

The patient was started on a therapeutic heparin infusion. Her BP was managed with nifedipine 60 mg extended release nightly and labetalol 200 mg every eight hours. The pulmonary edema was treated with 40 mg of furosemide. Her hyperglycemia was treated with an insulin infusion adjusted to an hourly assessment of her capillary glucose. Betamethasone 12 mg every 24 hrs was given for fetal lung maturation. An external monitor recorded uterine activity and the fetal heart rate. Multiple lab parameters were assessed every six hours. 

There was a multidisciplinary patient care conference, which included maternal fetal medicine, cardiology, pulmonary, interventional radiology, and neonatology, to discuss treatment of the PE with an embolectomy or continued therapeutic anticoagulation, as well as to develop a delivery plan. Since there was no evidence of right heart strain, the consensus was that fluoroscopic exposure and surgery for an embolectomy presented more risk than continuing the anticoagulation. Due to her pulmonary edema, the patient was advised that she should have a repeat cesarean section 24 hours after her second dose of betamethasone. 

On hospital day two, the patient improved with oxygen saturation of 97-99% on four liters of oxygen via nasal cannula, well-controlled BP, and a down-trending BNP from 445 to 413. Her ECHO showed preserved right ventricular function with RAP at 0-5 mm. A CT venogram showed a 1.3 x 0.6 cm filling defect immediately cranial to the apex of the IVC filter, demonstrating the known PE in the lower lobes and pulmonary edema with bilateral small pleural effusions. Since there was still preserved cardiac function, the therapeutic anticoagulation was continued. 

On hospital day four at 30 weeks and six day gestation, the patient had repeated severe range BPs, defined as systolic blood pressure of 160 mm Hgb or higher and/or diastolic blood pressure of 110 mm Hg taken 15 minutes apart, with physical exam finding significant for crackles in the left lung base and an increase in oxygen requirement to saturation above 95%. The aspartate aminotransferase (AST)/alanine aminotransferase (ALT) ratio increased from 34/29 (hospital day one) to 133/146, respectively, and the serum creatinine rose from 0.45 to 0.65.

The decision was made to proceed with a cesarean section under general anesthesia. The heparin infusion was held, protamine was available, and the patient was cross-matched for four units of packed red blood cells. The plan was to resume heparin six hours pos delivery. An uncomplicated repeat cesarean section with bilateral salpingectomy was performed with an estimated blood loss of 690 cc. The infant weighed 1620 g with Apgar scores of 4 at one minute and 8 at five minutes and was transferred to the neonatal intensive care unit.

The patient was reinitiated on a heparin drip six hours postoperatively. On postoperative day one, PTT was 100 and anti-Factor Xa was 1.4; therefore, the heparin drip was discontinued, and hematology oncology recommended transition to enoxaparin 1 mg/kg as a five-day bridge to therapeutic warfarin with a goal of an INR of 2-3. The remainder of her postoperative course was unremarkable, and the patient continued to improve. She was discharged on postoperative day six on therapeutic enoxaparin (120 mg every 12 hours) until therapeutic on warfarin 7.5 mg daily for four to five days, nifedipine 30 daily and 60 mg nightly, and labetalol 300 mg every eight hours. She was scheduled for follow-up appointments with hematology. 

## Discussion

VTE, which consists of PE and DVT, is a known and serious complication of pregnancy, accounting for 3.2% of all maternal deaths worldwide [7] and 15% of maternal deaths in the United States between 2003 and 2011 [8]. The risk of peripartum VTE is increased compared to nonpregnant women, with six weeks postpartum period having the highest day-to-day risk [3]. Thus, understanding risk factors, clinical presentation, diagnostic criteria, and treatment options for PE is of utmost importance in pregnancy. 

The increased risk of VTE in pregnancy is related to the physiological and anatomic changes of pregnancy, including hypercoagulability, an increase in venous stasis, and vascular injury, known as Virchow's triad [3]. Physiological changes include a 40% decrease in protein S, a doubling of the concentration of fibrinogen, a 20-100% increase in factors VII, VIII, IX, X, and XII, and an up to 400% increase in von Willebrand factor [9]. There is decreased venous outflow [10], compression of the IVC and pelvic veins by the enlarging uterus [11], and decreased mobility [12]. In addition, a decrease in anti-thrombin III levels has clinical implications, including an increased risk of thrombosis and thromboembolism [13]. 

Pregnant women have a nearly 10-fold increased risk of VTE compared to non-pregnant women of comparable age [9]. The current patient had multiple risk factors for a recurrent VTE. She had a personal history of thrombosis, the most important risk factor, as the risk of recurrence is increased three to fourfold, with 15-25% of cases being recurrent events [14]. The second most important individual risk factor is thrombophilia, and multiple family members had been diagnosed with a protein S deficiency, raising the concern that she, too, may be affected. In addition, studies have shown that low socioeconomic conditions, such as level of education, income, and occupational status, can increase the risk of thromboembolism during pregnancy, although the mechanisms are unclear [15,16]. 

PE can present in multiple ways, ranging from being asymptomatic to a sudden death. It is important to classify PE in order to guide management. The most commonly used classification systems are those proposed by the AHA [17] and the ESC [18]. Both divide PE severity into three main categories: massive (AHA) or high risk (ESC), submassive (AHA) or intermediate-risk (ESC), and low-risk (ESC and AHA). Massive or high risk is characterized by hypotension, defined as systolic BP <90 mm Hg, a drop of >40 mm Hg for at least 15 minutes, or need for vasopressor support. Submassive is characterized by AHA as RV strain without hypotension or evidence of RV injury by an increase in cardiac biomarkers such as troponin [17]. Intermediate-risk is defined by ESC as having a simplified Pulmonary Embolism Severity Index (PESI) score of ≥ 1 (i.e., oxygen saturation <90%), regardless of RV strain [18]. Low-risk PE does not meet the criteria for submassive or intermediate PE. 

The patient presented is unusual in that her PE would be classified as low-risk by AHA for the absence of RV strain, but as borderline intermediate-risk by ESC for oxygen saturation of 90%. Her PE was extensive, with occlusion of the left main pulmonary artery with resultant occlusion of all distal pulmonary arteries. Nevertheless, her PE was classified as low-risk for the absence of RV strain.

There is no single consensus to guide clinical decision making regarding routine pharmacologic thromboprophylaxis during and after pregnancy in the United States [18]. Rather, patients should be risk-stratified via personal and family history gathering. Given no single universal VTE risk assessment protocol, the American College of Obstetricians and Gynecologists (ACOG) recommends that each facility review its own protocols and adopt a single one in a systematic way to reduce the incidence of a VTE [19]. Adjusted dose anticoagulation is recommended with all women with acute VTE in pregnancy [20].

## Conclusions

PE is a serious complication of pregnancy that remains difficult to both diagnose and manage, especially in the setting of the physiological changes of pregnancy. Besides the range of clinical presentations of PE, other factors that complicate the diagnosis include the infrequency, comorbidities, and socioeconomic factors. Clinicians should have a higher index of suspicion, especially when patients have increased risk factors, and appropriately educate all patients on the signs and symptoms of PE.

Establishing the diagnosis effectively and quickly is imperative. Although there is no single consensus to guide clinical decision making for treating PE in pregnancy, taking an individualized approach, with multispecialty input, classifying the PE, and risk-stratifying are vital to management planning. This case demonstrates that in patients with multiple risk factors, including chronic hypertension with superimposed preeclampsia, poorly controlled diabetes, and possible protein S deficiency, anticoagulation alone may be sufficient management for large pulmonary emboli, as this approach decreases both maternal and fetal risk.

## References

[1] Abe K, Kuklina EV, Hooper WC, Callaghan WM (2019). Venous thromboembolism as a cause of severe maternal morbidity and mortality in the United States. Semin Perinatol.

[2] Cueto-Robledo G, Cervantes-Naranjo FD, Gonzalez-Hermosillo LM, Roldan-Valadez E, Graniel-Palafox LE, Castro-Escalante KY, Orozco-Zuñiga B (2023). Pulmonary embolism during pregnancy: an updated review with case series description. Curr Probl Cardiol.

[3] Dado CD, Levinson AT, Bourjeily G (2018). Pregnancy and pulmonary embolism. Clin Chest Med.

[4] Mehta LS, Warnes CA, Bradley E (2020). Cardiovascular considerations in caring for pregnant patients: a scientific statement from the American Heart Association. Circulation.

[5] Bates SM (2021). Pulmonary embolism in pregnancy. Semin Respir Crit Care Med.

[6] Giri J, Sista AK, Weinberg I (2019). Interventional therapies for acute pulmonary embolism: current status and principles for the development of novel evidence: a scientific statement from the American Heart Association. Circulation.

[7] Say L, Chou D, Gemmill A (2014). Global causes of maternal death: a WHO systematic analysis. Lancet Glob Health.

[8] Kuriya A, Piedimonte S, Spence AR, Czuzoj-Shulman N, Kezouh A, Abenhaim HA (2016). Incidence and causes of maternal mortality in the USA. J Obstet Gynaecol Res.

[9] Gabbe SG, Niebyl JR, Simpson JL (2017). Obstetrics: Normal and Problem Pregnancies. Landon MB, Galan HL, Jauniaux ERM, Driscoll DA, Berghella.

[10] Antony KM, Racusin DA, Aagaard K, Dildy III GA (2017). Maternal physiology. Obstetrics: Normal and Problem Pregnancies.

[11] Whitty JE, Dombrowski MP (2013). Respiratory diseases in pregnancy. Creasy and Resnik’s Maternal-Fetal Medicine: Principles and Practice.

[12] Danilenko-Dixon DR, Heit JA, Silverstein MD, Yawn BP, Petterson TM, Lohse CM, Melton LJ 3rd (2001). Risk factors for deep vein thrombosis and pulmonary embolism during pregnancy or post partum: a population-based, case-control study. Am J Obstet Gynecol.

[13] Bravo-Pérez C, Vicente V, Corral J (2019). Management of antithrombin deficiency: an update for clinicians. Expert Rev Hematol.

[14] Pabinger I, Grafenhofer H, Kyrle PA, Quehenberger P, Mannhalter C, Lechner K, Kaider A (2002). Temporary increase in the risk for recurrence during pregnancy in women with a history of venous thromboembolism. Blood.

[15] Park JE, Park Y, Yuk JS (2021). Incidence of and risk factors for thromboembolism during pregnancy and postpartum: a 10-year nationwide population-based study. Taiwan J Obstet Gynecol.

[16] Zöller B, Li X, Sundquist J, Sundquist K (2012). Socioeconomic and occupational risk factors for venous thromboembolism in Sweden: a nationwide epidemiological study. Thromb Res.

[17] Jaff MR, McMurtry MS, Archer SL (2011). Management of massive and submassive pulmonary embolism, iliofemoral deep vein thrombosis, and chronic thromboembolic pulmonary hypertension: a scientific statement from the American Heart Association. Circulation.

[18] Konstantinides SV (2014). 2014 ESC Guidelines on the diagnosis and management of acute pulmonary embolism. Eur Heart J.

[19] Sibai BM, Rouse DJ (2016). Pharmacologic thromboprophylaxis in obstetrics: broader use demands better data. Obstet Gynecol.

[20] (2018). ACOG practice bulletin no. 196: thromboembolism in pregnancy. Obstet Gynecol.

